# PubMed Labs: an experimental system for improving biomedical literature search

**DOI:** 10.1093/database/bay094

**Published:** 2018-09-18

**Authors:** Nicolas Fiorini, Kathi Canese, Rostyslav Bryzgunov, Ievgeniia Radetska, Asta Gindulyte, Martin Latterner, Vadim Miller, Maxim Osipov, Michael Kholodov, Grisha Starchenko, Evgeny Kireev, Zhiyong Lu

**Affiliations:** National Center for Biotechnology Information, National Library of Medicine, National Institutes of Health, 8600 Rockville Pike, Bethesda, MD, USA

## Abstract

PubMed is a freely accessible system for searching the biomedical literature, with ∼2.5 million users worldwide on an average workday. In order to better meet our users’ needs in an era of information overload, we have recently developed PubMed Labs (www.pubmed.gov/labs), an experimental system for users to test new search features/tools (e.g. Best Match) and provide feedback, which enables us to make more informed decisions about potential changes to improve the search quality and overall usability of PubMed. In addition, PubMed Labs features a mobile-first and responsive layout that offers better support for accessing PubMed from increasingly popular mobiles and small-screen devices. In this paper, we detail PubMed Labs, its purpose, new features and best practices. We also encourage users to share their experience with us; based on which we are continuously improving PubMed Labs with more advanced features and better user experience.

## Introduction 

As the biomedical literature grows at an exponential rate, the National Library of Medicine (NLM) has recently developed PubMed Labs (www.pubmed.gov/labs), an experimental system for users to test new features/tools and provide feedback, which enables us to make more informed decisions about potential changes to improve the search quality and overall usability of PubMed (www.pubmed.gov). The purpose of this paper is to make the scientific community aware of PubMed Labs as well as to provide them with an in-depth description. To this end, we compare the features of PubMed Labs with those in PubMed and its current mobile version (PubMed Mobile). In doing so, we show the differences between PubMed Labs and the current ones and how it is advanced in ultimately becoming PubMed 2.0 (1). We also demonstrate that all of the features contained in PubMed Mobile are already made available and enriched in PubMed Labs.

As we continue to develop PubMed Labs and add functionalities (new or revamped ones from PubMed), it is also important to show how to best use PubMed Labs. Some features, albeit already existing in PubMed, may have a new form in PubMed Labs, which we aim to describe comprehensively here. Finally, our last objective is to invite the broad research community to test PubMed Labs and, more importantly, to provide feedback. We hope to build PubMed 2.0 together with our users so they can have the optimal experience for biomedical literature search (2, 3).

PubMed Labs has several unique features that distinguish it from PubMed and other search systems for the biomedical literature (2, 4, 5). (i) By default, given a free-text query as input, search results are sorted by relevance in order to provide users with the most pertinent information (in PubMed, the default sort order is Most Recent) as suggested by previous research (6–10). This is made possible based on a newly developed cutting-edge relevance search algorithm called Best Match (11). In essence, Best Match relies on a state-of-the-art machine-learning algorithm trained on past user search history with many relevance signals, e.g. the popularity of an article, its publication date and type and query-document relevancy score (12). In addition, to help users identify articles of interest, search results include snippets, which are useful highlighted text fragments from the article abstract that are selected based on their relatedness with the user query. (ii) PubMed Labs has a more modern user interface. Users will find it easier to discover related content (e.g. similar articles, references and citations). (iii) Compatibility with smaller screen portable devices (e.g. phones, tablets and laptops) is optimized to ensure the best possible searching and reading experience on such devices. (iv) Finally, please note that by design PubMed Labs includes only a limited set of highly used features (13) and not the entire set currently available in PubMed. Based on public testing and feedback, we will iteratively include additional functions and improve the system towards PubMed 2.0 (1) over time. PubMed Labs was first made public in October 2017 and is currently accessed by thousands of users from around the world each day.

## Materials and methods

### Data indexing

As of 2018, there are over 28 million articles in PubMed Labs where each article is indexed via the following separate data fields: titles, abstracts, MeSH terms etc. Although the number of articles is identical in PubMed vs. PubMed Labs, the two systems make use of different indexing systems. In PubMed Labs, we use Solr, an open-source enterprise search system (http://lucene.apache.org/solr/), for document indexing and retrieval. In addition to its scalability and reliability, Solr also provides many out-of-the-box search functionalities, such as better understanding wildcards (‘^*^’). For example, because PubMed limits the number of variants for wildcards, the query therap^*^ and cell[jour] and 2017[year] only yields 77 hits in PubMed while 129 results are returned by Solr in PubMed Labs (accessed on 23 February 2018). Another notable Solr feature is to integrate synonyms during term indexing such that it results in significant improvements in search time. Finally, in PubMed Labs, the underlying document data for indexing is newly generated by merging content from PubMed, Books (the NLM’s digital archive of full text life sciences books and documents—of which excerpts are also found in PubMed) and PubMed Central (PMC) such that it allows the display of relevant information not available in PubMed (e.g. references from PMC).

### User interface infrastructure

PubMed Labs is a Django (https://www.djangoproject.com/) application on the front-end, making use of the latest web technologies and standards. It is compatible on any screen size and provides a fresh and consistent look and feel throughout the application.

### Integration of third-party analytics tools

PubMed Labs is first and foremost an experimental system; therefore, seeking and analysing user feedback is a critical component. To this end, we used a third-party analytic tool (Google Analytics) in order to gather aggregated user behaviours and trends. This provides a convenient way for us to investigate the utility of certain features and to determine which ones are more needed and vice versa. Additionally, we use Google Optimize to set up A/B tests, controlled experiments for comparing variants of certain features. The key idea of A/B testing is to study usage and interactions with variants (either functional or cosmetic) to better understand how features should be best implemented. For example, A/B testing was used to demonstrate that the new Best Match (11) algorithm returns more relevant results vs. the default date sort system in terms of user click through rate (39 vs. 32%, a relative increase of 22%). Our findings gathered from these quantitative usage studies are also supplemented with other types of user research such as user interviews as they provide complementary perspectives. Finally, please note that our use of Google Analytics strictly follows the National Library Medicine’s Web Security and Privacy Policy (https://www.nlm.nih.gov/privacy.html).

**Table 1:** Comparison of features available in PubMed, PubMed Mobile and PubMed Labs. The + sign denotes a significant improvement of the feature in PubMed Labs compared to the current PubMed. AMA, MLA and APA stand for citation formats American Medical Association, Modern Language Association and American Psychological Association, respectively

**Feature**	**PubMed**	**PubMed Mobile**	**PubMed Labs**
**Results by year (Figure 1d)**Bar chart with columns representing the number of articles for the entered query every year.	Yes	No	Yes+Possibility to select specific time periods
**Search facets (Figure 1e)**Filters to quickly narrow down the results (e.g. publication date).	Yes	Partially (10)	Partially (12)
**Snippets (Figure 1f)**Useful highlighted text fragments from the article abstract that are selected based on their relatedness with the user query.	No	No	Yes
**Related searches (Figure 1g)**Suggested queries close to the one entered by the user.	Yes	Yes	Yes
**Abstract format**Search results can be displayed with more information, e.g. abstract.	Yes	No	Yes+ Number of PMC citations, references and figures
**Article figures (Figure 2i)**Figures in the article when available.	Yes	No	Yes+Full screen carousel, better user interaction
**Citation data (Figure 2k)**An easy access to various citation formats (AMA, MLA, APA).	No	No	Yes
**Article navigation (Figure 2m)**List of sections of an article with a pointer on the current section.	No	No	Yes
**Similar articles (Figure 2n)**A list of articles related to the one displayed.	Yes	Yes	Yes+More details, more standing out
**Next/previous article**Links allowing to skim through the results without going back and forth to the search results page.	No	Yes	Yes
**Advanced search page**A page containing various tools (e.g. query builder, search history) to help more advanced users design their complex queries.	Yes	No	No
**MyNCBI**A collection of features (e.g. saving sets of articles) available after logging in.	Yes	No	No

**Figure 1:**
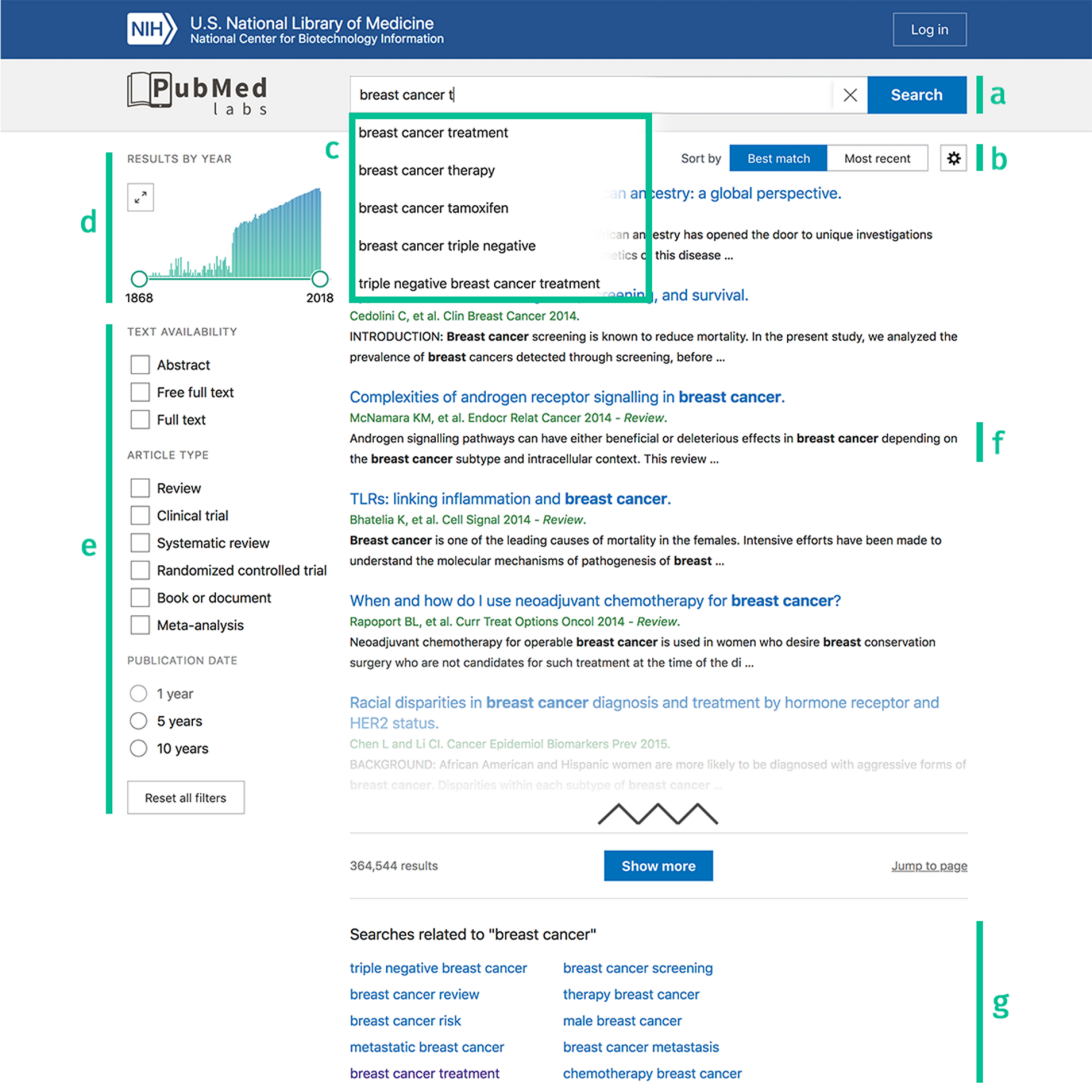
PubMed Labs’ search results page with highlighted features. **(a)** Search box. **(b)** Sort order toggle and display options. **(c)** Query auto completion. **(d)** Results by year. **(e)** Search facets. **(f)** Highlighted search terms in title and snippet. **(g)** Related searches.

### Best practices for using PubMed Labs 

There are a number of noticeable differences between PubMed Labs, the current PubMed and its mobile version (PubMed Mobile) with respect to user interactions and search experiences. A summary comparison of the most important features in PubMed and PubMed Mobile vs. PubMed Labs is shown in Table 1. We also describe them in details below.

### How to search

In order to run a search, users can type their queries made of free keywords in the search box (Figure 1a). As in PubMed, field tags e.g. ‘[author]’ can be attached to the queries with the same syntax conventions (https://www.ncbi.nlm.nih.gov/books/NBK3827/#pubmedhelp). Search Field Descriptions and Boolean operators (e.g. ‘AND’, ‘OR’, ‘NOT’) are supported and the query syntax remains the same. For sort orders, PubMed Labs currently supports the two most used ones in PubMed: (i) Best Match and (ii) Most Recent. By default, results are retrieved using the Best Match sort order as it aims to return the most relevant information given a query. The Best Match algorithm is built on a state-of-the-art machine-learning approach and incorporates many relevance signals to find the most pertinent information at the top of the returned results. Meanwhile, some users’ needs may be better served with the Most Recent sort order (e.g. browsing the latest issue of a journal). Thus, in PubMed Labs the two sort orders are displayed next to each other and can be switched easily (Figure 1b) with a single click (PubMed Labs also remembers the last sort order users chose to use). We believe the single-click switch is also convenient for users to compare results provided by both sort orders in some use cases. Note that query auto completion and related searches are also available (Figure 1c and 1g) as in PubMed. Finally, we have recently developed a tool called field sensor towards natural language understanding and semantic search (14) where it is deployed to identify queries consisting only of author names or journal names (e.g. ‘nature’). When detected, the date sort order is automatically activated for ranking search results as relevancy is less applicable in such search scenarios.

### How to examine search results

If a search returns only one result, PubMed Labs displays the article abstract page, the same behaviour as PubMed. For all other searches, a summary of search results is displayed (Figure 1). This page is critical in all search engines as it allows the users to quickly get an idea of the results returned. Hence, this page needs to provide enough information for users to judge which articles might satisfy their information need while not overwhelming them. For PubMed Labs, this page includes both traditional as well as new features.

**Figure 2:**
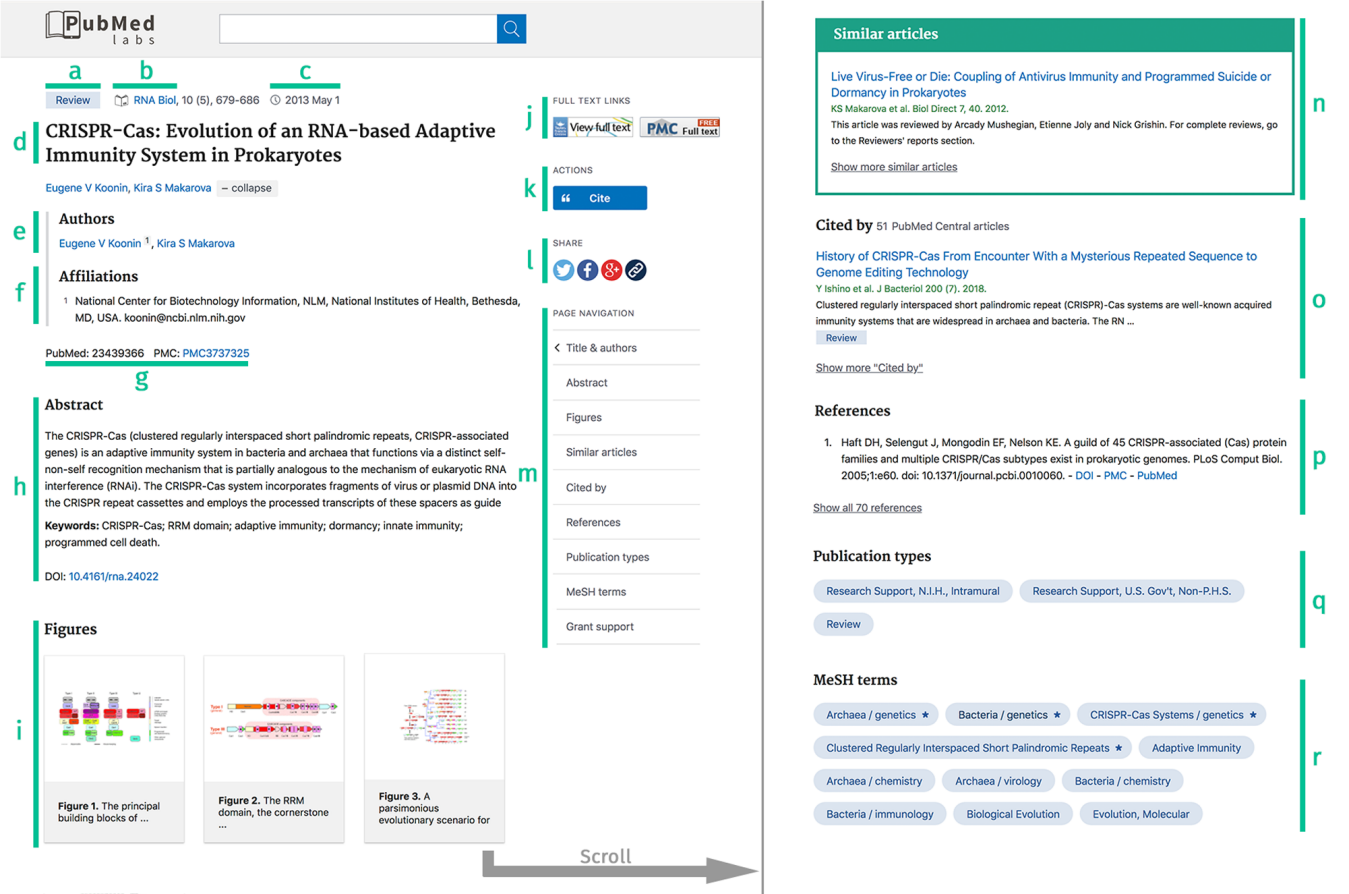
PubMed Labs abstract page with highlighted content. **(a)** Major publication type. **(b)** Abbreviated journal name. **(c)** Publication date. **(d)** Title. **(e)** List of full author names. **(f)** Author affiliations. **(g)** PMID and PMCID. **(h)** Abstract. **(i)** Figures. **(j)** Full text links. **(k)** Cite button. **(l)** Sharing options. **(m)** Navigation menu. **(n)** Similar article list. **(o)** List of articles citing this paper in PMC. **(p)** References listed in this article. **(q)** Full list of publication types. **(r)** MeSH concepts indexing this article.

#### Article details and snippets

In PubMed Labs, only the top 10 results are displayed in the results page, compared to 20 in PubMed. However, the matching query terms are highlighted for each result in PubMed Labs to help users better understand why they were returned. This would be especially useful if an article matched a synonymous term in the query. For each returned article, this author list has been shortened for added consistency and readability in the results page. Up to two authors are listed, and articles with more than two authors are shown with the name of the first author followed by ‘et al.’. However, if a query contains an author name (e.g. Koonin E.V.), matching articles will always highlight the author name in the results.

The journal name and the article type (if it is a review or a clinical trial) are provided next to the author list. PubMed Labs also brings an important new feature compared to PubMed by showing snippets, which are excerpts from abstracts that best match the query and provide additional contextual information. This helps to show how the returned article is related to the search query.

#### Results by year

A bar chart (Figure 1d) shows the number of publications in a given year. While this feature already exists in PubMed, it has been significantly enriched to better meet the users’ needs. For example, the widget can now be extended to take the full-page width by clicking the top left icon, and the two handles can be dragged to select specific time periods.

#### Search facets

As in PubMed, search facets are displayed on the left to enable users to easily refine their search(Figure 1e) and narrow down search results. While this list is less comprehensive than its PubMed counterpart, these facets are the most used ones and should satisfy most of the needs while we work on adding more. Text availability and article type can be combined (e.g. by selecting both ‘Abstract’ and ‘Full text’, which will return articles matching any of the two), while only one publication date facet can be selected at a time. Note that ‘Full text’ differs from ‘Free full text’ in such a way that ‘Full text’ simply indicates that there is a link available to the full text but that might require user log in or pay for the article, while ‘Free full text’ indicates that full text is freely available without any access restrictions (e.g. full-text articles in PMC). This difference, however, does not impact the search results in PubMed Labs as only title and abstracts are indexed and used for retrieval currently. The selected filters are saved within a search sequence (i.e. during refinements and back and forth with articles). A reset button at the bottom allows users to conveniently remove all filters associated with the search with a single click.

### How to examine each individual article 

The abstract page, displayed after a click on an article title in the search results page, is another critical component of our system. It provides more details about the article and includes rich information for the user to decide in their next step (e.g. downloading the full text, refining the query, browsing similar articles etc).

#### Publication metadata

The top of our abstract page is dedicated to the article’s metadata. Particularly, as detailed in Figure 2, it provides (a) the publication type, (b) journal name, (c) publication date, (d) title, (e) list of author names, (f) their affiliations and (g) the article’s PubMed ID (PMID) and PubMed Central ID (PMCID). The main difference is that we display full author names in PubMed Labs rather than just initials and last name as in PubMed.

#### Abstract and figures

Right underneath in the main area of the page lay the article contents, i.e. the abstract (Figure 2h) and figures (Figure 2i), when available. The figure display has been entirely revamped to provide a cleaner and more user-friendly look, and it features an in-page full-size view that you can share simply by copying the URL.

#### Similar articles

Another highly used feature in the PubMed abstract page is the similar articles. These suggestions are calculated for every article (15) and the most similar ones can provide important and interesting additional information. In PubMed Labs, the title, first author, journal name, publication year and first two lines of the abstract are displayed for the five most similar articles.

#### Citation data

Two types of references are displayed next (i) cited by and (ii) article references. The former shows only articles available in PMC that are citing this article. The latter is the entire list of references with links to PubMed and PMC, when available. Both are conditioned by data availability so not all articles have this information.

#### Next/previous article

As in PubMed Mobile, PubMed Labs now shows next and previous buttons at the very bottom of the page, displayed in the results page format. When used on small-screen devices (e.g. phones), PubMed Labs also shows these buttons at any scrolling position, with a quick glance at the articles when hovered over.

#### Right column

As usual, full text links are displayed at the top right position of the screen. Note, however, that this column is now made sticky, meaning that scrolling does not make it disappear. Moreover, a new cite button is shown, which allows the users to have easy access to citation information in AMA, MLA and APA format. The citation can also be downloaded in RIS format, useful for citation managers. Finally, the right column features a navigation menu that allows users a quick glance at what associated content is available for the article.

### Use cases and usage statistics 

Since PubMed Labs includes nearly all features of PubMed Mobile and that it is responsive to small screen devices, we started inviting users on PubMed Mobile to try out PubMed Labs with a promotion banner displayed on the PubMed Mobile website. As of April 2018, on an average weekday, there are over 3000 users from around the globe with ∼5000 searches and 7000 page views. Both sort orders are used by our users: Best Match (94%) and Most Recent (6%). As in PubMed, the most popular user activities after reading the abstract are to retrieve the full text and/or read similar articles.

**Figure 3:**
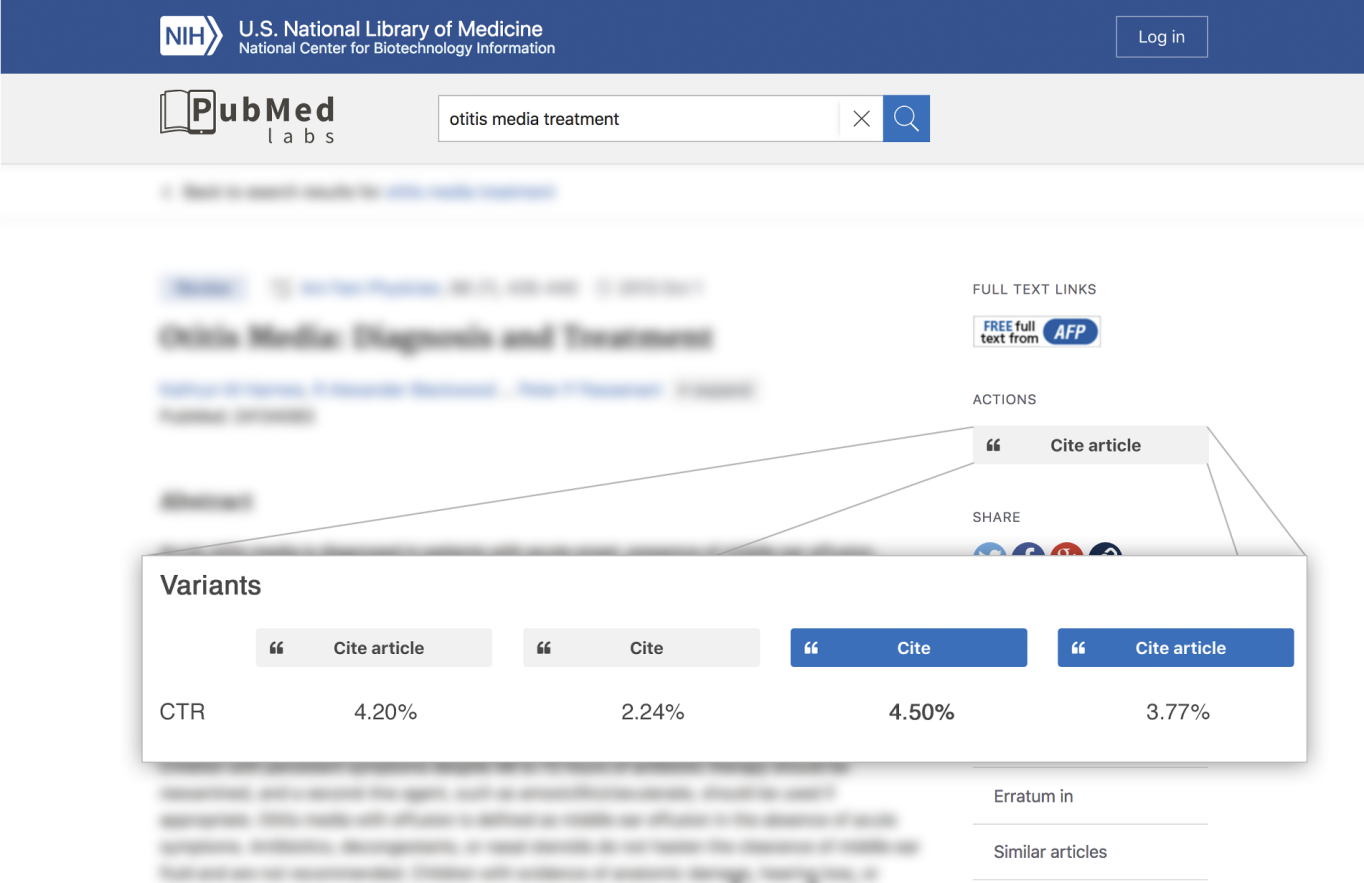
An experiment currently running on PubMed Labs where variants of the cite button design are being tested with their associated click-through rates.

**Figure 4:**
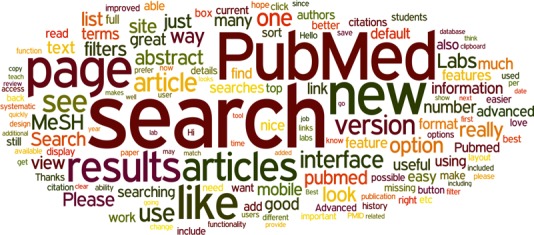
A word cloud representing the most popular words contained in the feedback comments on PubMed Labs.

Given its experimental nature, we have also started performing various A/B testing to refine the interface and certain features in PubMed Labs. As a simple example, we have compared four variants for the newly created cite button in the abstract page (Figurue 3). The basic version has the background colour in grey with the text ‘Cite’. Alternatively, we have tested the combinations of another background colour (blue) as well as a different wording (‘Cite article’) for this. The results indicated that ‘Cite’ with a blue background is the most preferred option by our users.

Moreover, we implemented a feedback button on all PubMed Labs pages and we have received more than 200 comments since its official release. Figure 4 shows a graphical summary of their contents. As can be seen, the feedback from our users is positive overall (‘like’, ‘great’, ‘thanks’); they like the new search and how easy it is to use the new interface. Meanwhile, they also inquired about missing features (e.g. advanced search, MyNCBI) and whether they will be integrated eventually, which we are already aware of and we will add them for optimal user experience.

### Summary and future directions 

As an experimental system, PubMed Labs provides opportunities to experiment and innovate within a powerful sandbox to advance the limits of literature search and gather user feedback. PubMed Labs currently includes a subset of core functionalities of PubMed (search, major sort orders, most used facets etc.) as well as a number of new experimental features (e.g. snippets or the cite button). Despite that, its functionality is limited compared to PubMed where it offers additional features such as advanced search, MyNCBI etc. A future direction is to iteratively add and test new features based on user input. We strongly encourage our users to continue testing PubMed Labs and sharing their experience with us, which complements the insight we can glean from usage analysis and other user research. With the help from our users, we hope to jointly improve PubMed Labs with more features and better user experience in the future, and we expect to turn it into the new PubMed in the near future, first with its mobile users and ultimately to all users.

## Notes

Database URL: https://www.pubmed.gov/labs
